# Supplementary material for: Comparative Study on Macro-Tribological Properties of PLL-g-PEG and PSPMA Polymer Brushes
Source: Polymers (Basel). 2022 May 8;14(9):1917. doi: 10.3390/polym14091917 (PMC9103741; doi:10.3390/polym14091917)
Supplement: Supplementary file 1 [file polymers-14-01917-s001.zip › polymers-1684891-supplementary.pdf]

Supplementary Materials for

Comparative study on macro-tribological properties of PLL-g-PEG and PSPMA polymer brushes

Fue Ren [1], **Shuyan Yang [1,*], Yang Wu [2], Feng Guo [1], and Feng Zhou [2,*].**

*Correspondence: yangshuyan@qut.edu.cn; zhouf@licp.cas.cn.

This file includes: Figures S1–S6

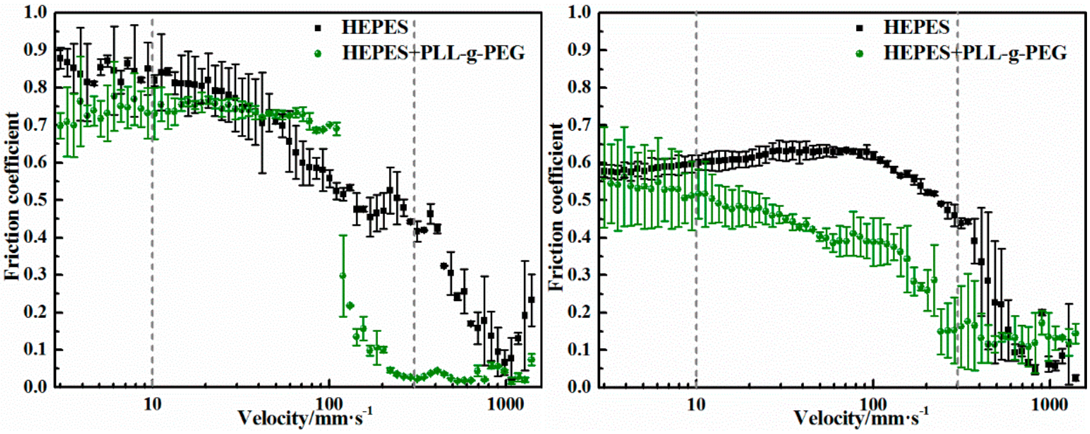

(a) Load = 0.5N (b) Load = 3.5 N

Figure S1. Stribeck curves lubricated by HEPES and HEPES added by PLL-g-PEG at the load of 0.5N and 3.5N, respectively. The Stribeck curves was obtained from 1400 mm/s to 3 mm/s, 10 mm/s and 300 mm/s was selected to study the effect of PLL-g-PEG on friction at full-film hydrodynamics and boundary lubrication regime, respectively.

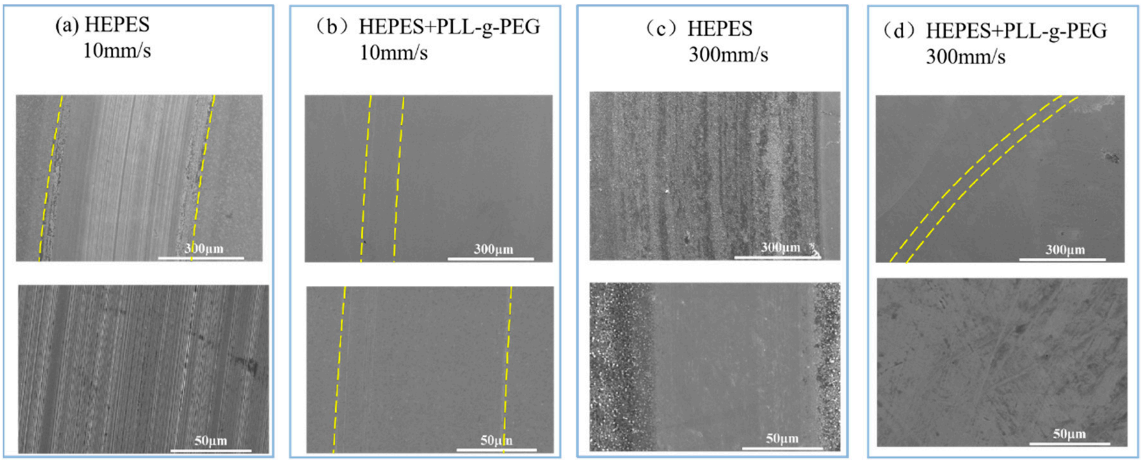

Figure S2. SEM image of wear scar on steel ball after friction testsrat the load of 0.5N: indicating the reduction in wear by adding PLL-g-PEG.

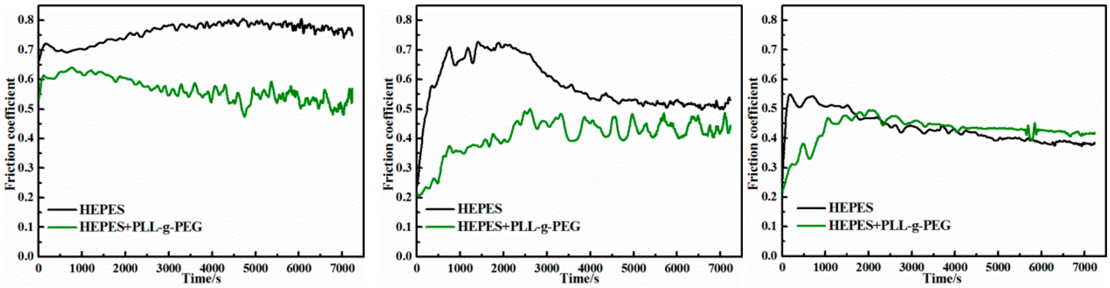

(a) Glass ball-glass plate (b) Steel ball-glass plate (c) Steel ball-steel plate.

Figure S3. Curves of PLL-g-PEG on friction on different substrate at the load of 0.5N and the

velocity of 10mm/s, indicating that the effect of the polymer brush on friction was greatly affected by the base material due to the wettability of the surface.

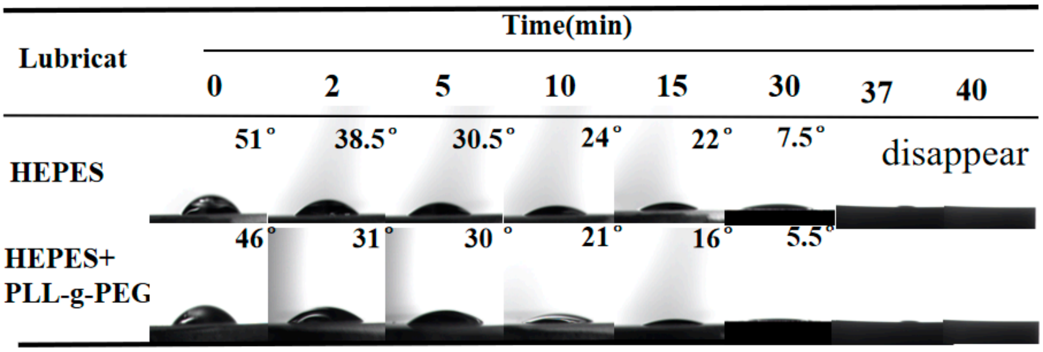

(a) On steel substrate

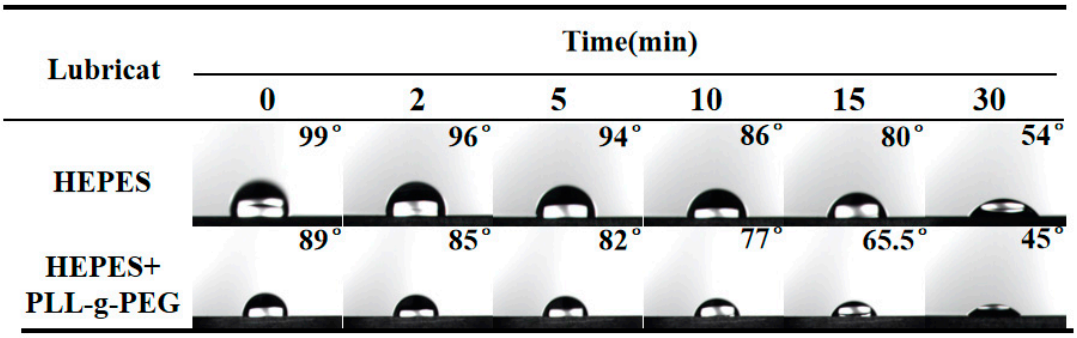

(b) On glass substrate

Figure S4. Contact angles on steel and glass substrate lubricated by HEPES and HEPES added by PLL-g-PEG, showing that contact angle decreases gradually with time and basically become stable after 30 minute.

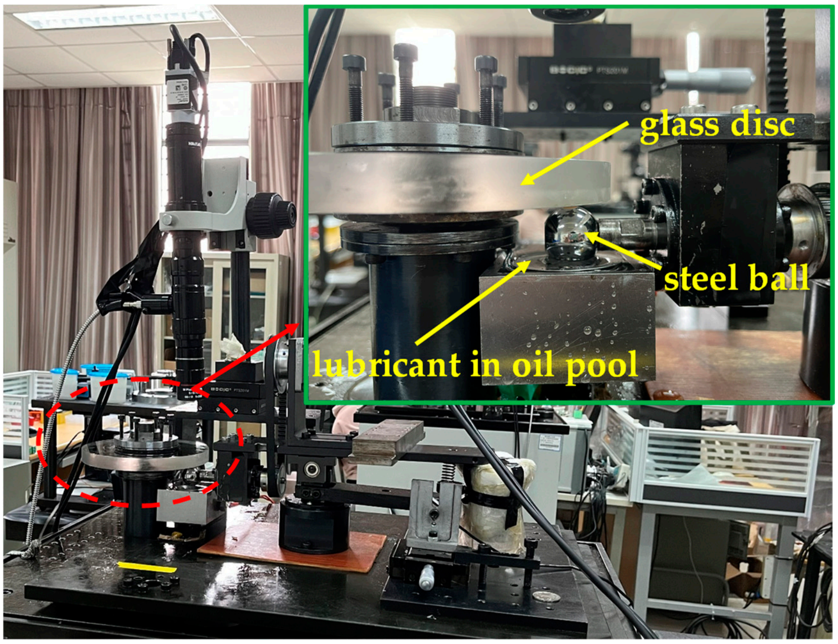

Figure S5. Physical diagram of laboratory set for measuring the film thickness, the oil pool with 10 μL of lubricants for a fully flooded (EHL) condition.

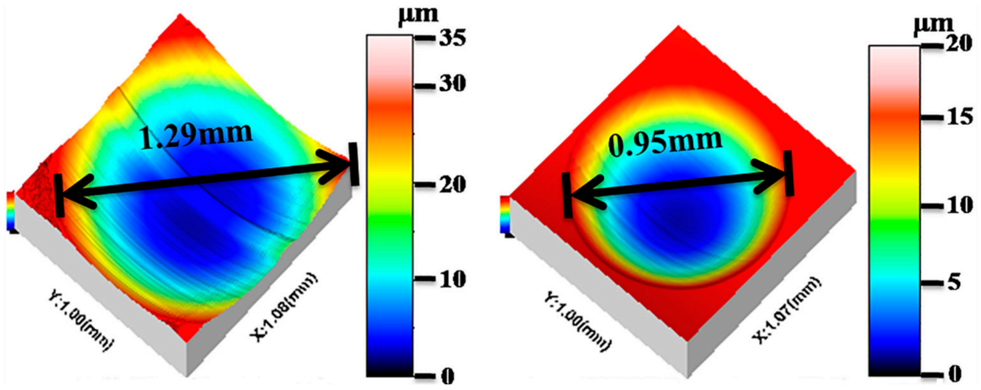

(a) HEPES, 10mm/s (b) HEPES added by PLL-g-PEG, 10mm/s

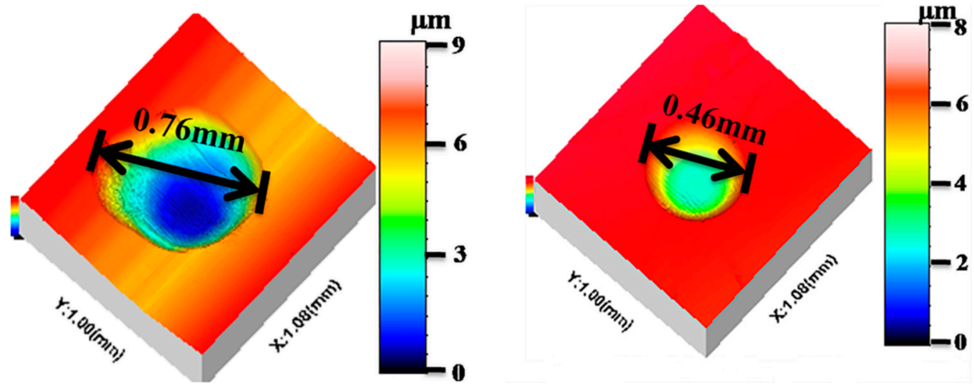

(c) HEPES, 300mm/s (d) HEPES added by PLL-g-PEG, 300mm/s

Figure S6. Topography of wear scar on glass plates after friction tester using Form Talysurf PGI 800(Taylor Hobson Co. Ltd., Leicester, UK), showing smaller diameter of wear scar by adding PLL-g-PEG. Form
